# Clinical Impact of Graft Cryopreservation on Allogeneic Stem Cell Transplantation: An Italian, Registry‐Based Study on Behalf of the “Gruppo Italiano Per Il Trapianto di Midollo Osseo, Cellule Staminali Emopoietiche e Terapia Cellulare” (GITMO)

**DOI:** 10.1002/ajh.27731

**Published:** 2025-06-02

**Authors:** Irene Defrancesco, Virginia Valeria Ferretti, Patrizia Chiusolo, Domenico Russo, Chiara Nozzoli, Attilio Olivieri, Massimiliano Gambella, Irene Maria Cavattoni, Stefania Bramanti, Stella Santarone, Renato Fanin, Roberto Cairoli, Simona Piemontese, Matteo Parma, Francesco Onida, Alessandro Busca, Luca Castagna, Angela Cuoghi, Domenico Pastore, Nicola Mordini, Fabio Benedetti, Cristina Skert, Carlo Borghero, Anna Paola Iori, Franca Fagioli, Vincenzo Pavone, Carmine Selleri, Simone Cesaro, Maurizio Musso, Marco Ladetto, Daniele Vallisa, Paola Carluccio, Alessandra Picardi, Monica Tozzi, Alessandra Biffi, Giuseppe Milone, Maura Faraci, Arcangelo Prete, Lucia Prezioso, Antonio Maria Risitano, Francesco Paolo Tambaro, Veronica Tintori, Piero Galieni, Fabrizio Pane, Caterina Zerbi, Antonio Bianchessi, Giulia Losi, Francesco Romano, Alessia Taurino, Elena Oldani, Nicola Polverelli, Francesca Bonifazi, Massimo Martino

**Affiliations:** ^1^ Unit of Bone Marrow Transplantation and Cellular Therapies Division of Hematology, Fondazione IRCCS Policlinico san Matteo Pavia Italy; ^2^ Department of Clinical‐Surgical, Diagnostic and Pediatric Sciences University of Pavia Pavia Italy; ^3^ Biostatistics and Clinical Trial Center Fondazione IRCCS Policlinico san Matteo Pavia Italy; ^4^ Department of Hematology Università Cattolica del Sacro Cuore Rome Italy; ^5^ Unit of Blood Diseases and Stem Cell Transplantation Department of Clinical and Experimental Sciences, University of Brescia, ASST Spedali Civili of Brescia Brescia Italy; ^6^ Department of Cellular Therapies and Transfusion Medicine Careggi University Hospital Florence Italy; ^7^ Clinica di Ematologia e Clinica Medica Università Politecnica Delle Marche Ancona Italy; ^8^ UOC Ematologia e Trapianto di Midollo Osseo IRCCS Ospedale Policlinico San Martino Genova Italy; ^9^ Divisione di Ematologia Ospedale di Bolzano Bolzano Italy; ^10^ IRCCS Istituto Clinico Humanitas Rozzano Italy; ^11^ Hematology Unit Department of Oncology and Hematology, Pescara Hospital Pescara Italy; ^12^ Hematology and Bone Marrow Transplantation Unit Azienda Sanitaria Universitaria Friuli Centrale Udine Italy; ^13^ Department of Hematology ASST Grande Ospedale Metropolitano Niguarda, Niguarda Cancer Center Milan Italy; ^14^ Department Medicine and Surgery Università Degli Studi di Milano Bicocca Milan Italy; ^15^ IRCCS Ospedale San Raffaele, Vita‐Salute San Raffaele University Milan Italy; ^16^ Department of Haematology and Bone Marrow Transplantation Unit Fondazione IRCCS San Gerardo Dei Tintori Monza Italy; ^17^ Haematology and BMT Unit ASST Fatebenefratelli Sacco, University of Milan Milan Italy; ^18^ Stem Cell Transplant Center AOU Città Della Salute e Della Scienza di Torino Turin Italy; ^19^ BMT Unit AOR Villa Sofia‐Vincenzo Cervello Palermo Italy; ^20^ Section of Hematology Department of Medical and Surgical Sciences, Azienda Ospedaliero‐Universitaria di Modena, University of Modena and Reggio Emilia Modena Italy; ^21^ Haematology Ospedale A. Perrino Brindisi Italy; ^22^ Az. Ospedaliera S. Croce e Carle Cuneo Italy; ^23^ Hematology and Stem Cell Transplantation Azienda Ospedaliera Universitaria di Verona Verona Italy; ^24^ Unit of Haematology and Bone Marrow Transplantation Ospedale Dell'angelo Venezia Mestre Italy; ^25^ Hematology Unit Department of Clinical Oncology, San Bortolo Hospital Vicenza Italy; ^26^ Hematology, Department of Hematology Oncology and Dermatology, AOU Policlinico Umberto I, Sapienza University of Rome Roma Italy; ^27^ Department of Public Health and Paediatrics University of Turin Turin Italy; ^28^ Paediatric Onco‐Haematology, Stem Cell Transplantation and Cellular Therapy Division Regina Margherita Children's Hospital, City of Health and Science of Turin Turin Italy; ^29^ Hematology and Bone Marrow Transplantation Ospedale C. Panico Tricase Italy; ^30^ Hematology Unit University Hospital “San Giovanni di Dio e Ruggi d'Aragona” Salerno Italy; ^31^ Pediatric Hematology Oncology Department of Mother and Child, Azienda Ospedaliera Universitaria Integrata Verona Verona Italy; ^32^ Department of Oncology, Onco‐Hematology Unit and TMO U.O.C Palermo Italy; ^33^ Hematology Unit Azienda Ospedaliera Di Alessandria Alessandria Italy; ^34^ Hematology Unit Department of Oncology and Hematology, Guglielmo da Saliceto Hospital Piacenza Italy; ^35^ Unit of Hematology and Stem Cell Transplantation AOUC Policlinico Bari Italy; ^36^ UOSC Ematologia Con Trapianto CSE AORN A. Cardarelli Naples Italy; ^37^ Stem Cell Transplant and Cellular Therapy Unit University Hospital of Siena Siena Italy; ^38^ Paediatric Hematology, Oncology and Stem Cell Transplant Division Maternal and Child Health Department, Padova University and University Hospital Padua Italy; ^39^ Division of Hematology and Bone Marrow Transplant Unit Azienda Ospedaliero Universitaria Policlinico “G.Rodolico‐San Marco” Catania Italy; ^40^ Hematopoietic Stem Cell Transplantation Unit Department of Hematology and Oncology, IRCCS Institute G. Gaslini Genoa Italy; ^41^ Pediatric Hematology and Oncology IRCCS Azienda Ospedaliero‐Universitaria di Bologna Bologna Italy; ^42^ Haematology and BMT Unit Azienda Ospedaliero‐Universitaria di Parma Parma Italy; ^43^ Hematology Unit Hospital “S. Giuseppe Moscati” Avellino Italy; ^44^ Stem Cell Transplantation and Cell Therapy Unit AORN Santobono Pausilipon Naples Italy; ^45^ Department of Pediatric Hematology/Oncology and Hematopoietic Stem Cell Transplantation AOU Meyer Florence Italy; ^46^ UOC Ematologia e Terapia Cellulare Ospedale C. E G. Mazzoni Ascoli Piceno Italy; ^47^ Hematology Unit Department of Medicine and Surgery, University of Naples “Federico II” Naples Italy; ^48^ Department of Molecular Medicine University of Pavia Pavia Italy; ^49^ Department of Oncology and Hematology Azienda Socio‐Sanitaria Territoriale Papa Giovanni XXIII Bergamo Italy; ^50^ IRCCS Azienda Ospedaliero‐Universitaria di Bologna Bologna Italy; ^51^ Stem Cell Transplantation and Cellular Therapies Unit (CTMO), Grande Ospedale Metropolitano “Bianchi‐Melacrino‐Morelli” Reggio Calabria Italy

**Keywords:** COVID‐19, cryopreservation, engraftment, graft versus host disease, hematopoietic stem cell transplantation

## Abstract

The coronavirus disease 2019 (COVID‐19) pandemic created major challenges for allogeneic hematopoietic stem cell transplantation (allo‐HSCT). Scientific societies and authorities recommended cryopreserving grafts before starting conditioning regimens, despite limited data on the clinical impact. The Italian Group for Bone Marrow Transplantation (GITMO) conducted a registry‐based study involving 3492 patients who underwent allo‐HSCT between March 2018 and September 2021. The cryopreserved cohort (*n* = 976) included patients who received cryopreserved grafts during the pandemic and was compared to the historical cohort (*n* = 2516). Graft cryopreservation was associated with a lower day 30 incidence of neutrophil and platelet engraftment (adjusted sHR = 0.8 and 0.7, *p* = 0.031 and *p* < 0.001, respectively) and delayed hematopoietic recovery. However, primary graft failure rates at day +30 were similar in the cryo and historical cohort (4% vs. 5%, respectively; *p* = 0.337), also after adjustment (RR = 1.19, *p* = 0.518). Day 100 incidence of grade II‐IV acute GVHD was comparable between the two groups (adjusted sHR = 1.2, *p* = 0.194). Regarding chronic GVHD incidence, we found that it was higher in patients aged < 18 years in the cryo group (adjusted sHR = 3.9, *p* = 0.002), but lower in those aged 18–55 years (adjusted sHR = 0.7, *p* = 0.008). Cumulative incidence of relapse did not differ between historical and cryo cohort (adjusted sHR 1.0. *p* = 0.943), as well as non‐relapse mortality (adjusted sHR 1.1, *p* = 0.196) and relapse‐free survival (adjusted sHR = 1.1, *p* = 0.197). However, a shorter overall survival was observed in the cryopreserved group (adjusted HR = 1.2, *p* = 0.038). Transplant centers should carefully balance the benefits and drawbacks of cryopreservation in allo‐HSCT.

## 1 Introduction

The coronavirus disease 2019 (COVID‐19) pandemic caused a serious healthcare crisis that undermined transplantation center's ability to infuse fresh donor cells into the intended recipients on the scheduled day of allogeneic hematopoietic stem cell transplantation (allo‐HSCT). At the time of the pandemic (March 2020), the scientific societies, donor registries, and regulatory authorities worldwide formally recommended to cryopreserve grafts before starting the conditioning regimen, although data about the clinical impact of cryopreserved grafts in allo‐HSCT were still scanty. Indeed, allografts cryopreservation has been associated with controversial results. Several studies agreed that patients receiving allo‐HSCT from cryopreserved grafts experienced delayed hematopoietic engraftment, although this effect was modest in most cases [1, 2, 3, 4, 5, 6, 7, 8, 9]. Conversely, the effect of allografts cryopreservation on post‐transplant outcomes such as acute and chronic graft‐versus‐host disease (GVHD) incidence, incidence of relapse, non‐relapse mortality, relapse‐free survival, and overall survival were discordant. Some studies have shown a higher incidence of acute GVHD (aGVHD) after allo‐HSCT from cryopreserved grafts [4, 5], whereas other studies reported similar incidence [3, 6, 10, 11, 12] with a lower rate of chronic GVHD (cGVHD) [3, 4, 5]. Moreover, cryopreservation may also impair the graft‐versus‐leukemia effect, as a higher relapse incidence and inferior relapse‐free survival have been found in a few studies [3, 5, 9]. The impact on non‐relapse mortality is still uncertain, while a detrimental effect of cryopreservation on overall survival has been described by some authors [4, 5, 9]. Most studies exhibited heterogeneity in terms of period, sample size, disease type, patient population, or were based on single‐center experiences.

To clarify these unmet clinical needs, the “Gruppo Italiano per il Trapianto di Midollo Osseo, cellule staminali emopoietiche e terapia cellulare” (GITMO) carried out a comprehensive registry‐based study, evaluating the safety and clinical effects of cryopreservation on engraftment and post‐transplant outcomes.

## 2 Study Design

This is a registry‐based study conducted on behalf of the GITMO, with participation from 44 adult and pediatric Transplant Centers (Table S1).

The principal objectives were to evaluate the safety of allografts cryopreservation and the impact of cryopreservation on hematopoietic engraftment, graft failure incidence, cumulative incidence of acute and chronic graft‐versus‐host disease (aGVHD and cGVHD, respectively), cumulative incidence of relapse (CIR), non‐relapse mortality (NRM), relapse‐free survival (RFS), and overall survival (OS). The impact of transit time (< 2 days vs. ≥ 2 days), defined as the interval between the collection date and the cryopreservation date, was also considered within patients who received allo‐HSCT from cryopreserved graft.

To compare the outcomes of cryopreserved and non‐cryopreserved grafts, we included all allo‐HSCT from any indication, donor, and stem cell source (excluding cord blood transplants) performed by GITMO Centers from March 1, 2018, to September 30, 2021. Since cryopreservation was extremely rare before the COVID‐19 pandemic [13, 14] we assumed that all transplants performed before March 2020 used non‐cryopreserved products. For patients transplanted from March 1, 2020, to September 30, 2021, only cryopreserved transplants were considered. This information was confirmed through a specific questionnaire sent to all participating transplant centers. Additional data concerning the hematopoietic stem cells (HSCs) collection, graft manipulation, cryopreservation date, cell dose of the cryopreserved grafts, and infusion adverse effects of cryopreserved products were also recorded.

All the clinical data collected were extracted by the GITMO registry, and all patients gave a formal consent for data collection, as previously reported [15]. A total of 3492 patients were included in the present analysis, with 976 in the cryopreserved group (“cryo cohort”) and 2516 in the non‐cryopreserved group (“historical cohort”).

The study was conducted according to the Declaration of Helsinki.

### 2.1 Statistical Analysis

Categorical variables were presented as counts and percentages of each category. Quantitative variables were summarized as median and interquartile range (IQR, 25th–75th percentile). The association between two categorical variables was assessed via Fisher's exact test. The Mann–Whitney test for independent samples was used to compare quantitative variables among two groups of patients. The reverse Kaplan–Meier method was used to assess the median follow‐up of the two cohorts.

Cumulative incidence (CI) of hematopoietic engraftment at day 30 (30‐day CI) after allo‐HSCT was the primary study outcome. Neutrophil engraftment was defined as the first of 3 consecutive days with an absolute neutrophil count > 0.5 × 10^9^/L. Platelet engraftment was defined as the first of 7 consecutive days with a platelet count ≥ 20 × 10^9^/L without platelet transfusion. Primary graft failure was defined, according to the established criteria [16], as the failure to achieve the pre‐specified neutrophil and platelet thresholds by day +30 following allo‐HSCT. To account for early deaths, primary graft failure was analyzed including only patients who were alive at day +30 (primary analysis) and then considering patients who died before day +30 as experiencing primary graft failure (sensitivity analysis).

Secondary outcomes included: cumulative incidence of aGVHD, cGVHD, relapse (CIR), non‐relapse mortality (NRM), relapse‐free survival (RFS), and overall survival (OS). Cumulative incidence of aGVHD or severe aGVHD was defined as the time between allo‐HSCT and the occurrence of a grade ≥ II aGVHD or grade III–IV aGVHD, respectively. Cumulative incidence of cGVHD or extensive cGVHD was defined as the time between allo‐HSCT and the occurrence of a cGVHD or of extensive cGVHD, respectively. Acute and chronic GVHD were diagnosed and graded as previously described [17].

Relapse‐free survival was defined as the time from allo‐HSCT to clinical evidence of relapse, progression, or death from any cause or last documented follow‐up. Non‐relapse mortality was defined as the deaths not related to the underlying hematological disease. Overall survival was defined as the time from allo‐HSCT to death from any cause or last documented follow‐up.

The cumulative incidence of neutrophil and platelet engraftment, aGvHD, cGVHD, CIR, and the NRM was estimated by the Kalbfleisch and Prentice method. For these analyses, death without the event of interest was considered a competing event. OS and RFS were calculated according to the Kaplan–Meier method.

For all outcomes, univariable and multivariable analyses were performed to estimate the impact of cryopreservation on post‐transplant outcomes. The multivariable analysis was conducted by adjusting for the following factors: age (< 18 years vs. 18–55 years vs. > 55 years), HLA match (haploidentical vs. matched related donor [MRD] vs. unrelated donor [URD]), hematopoietic stem cell source (peripheral blood [PB] vs. bone marrow [BM]), myeloablative conditioning (yes/no), disease status at allo‐HSCT (complete remission/not complete remission), T‐cell depletion (yes/no), Karnofsky score (90–100 vs. < 90), and at least one comorbidity (yes/no). Specifically, the Fine & Gray regression model for competing risk was used to assess the effect of cryopreservation on the following study outcome: cumulative incidence of neutrophil and platelet engraftment, CIR, NRM, cumulative incidence of aGVHD and cGVHD. Results from Fine & Gray regression models were reported in terms of sub‐hazard ratio (sHR) with its 95% confidence interval (95% CI). The effect of cryopreservation on primary graft failure was evaluated by generalized linear models for the binomial family, and results were reported as risk difference (RD) with 95% CI. The proportional hazard Cox regression model was used to compare OS and RFS of cryopreserved grafts to the historical cohort. Results from Cox regression models were reported as hazard ratio (HR) with its 95% CI. Both for Fine & Gray and Cox models, clustered sandwich standard errors have been calculated to account for the intra‐center correlation.

For all outcomes, the interaction term between cohort (cryopreserved vs. historical cohort) and age class was tested; in the case of a significant interaction (e.g., cGVHD), models were performed separately for age class.

Univariable and multivariable analyses were performed to assess the impact of transit time (< 2 days vs. ≥ 2 days), defined as the interval between the collection date and the cryopreservation date, on the study endpoints within the cryo cohort. In case of significant interaction between transit time and type of donor (URD vs. other), two separate univariable and multivariable models were developed, applying the same adjustment variables.

A *p* < 0.05 was considered statistically significant. Statistical analyses were performed using Stata 18 (StataCorp. 2023. *Stata Statistical Software: Release 18*. College Station, TX: StataCorp LLC.).

## 3 Results

### 3.1 Patients' Characteristics

Patients' characteristics are depicted in Table 1. Recipients of cryopreserved allografts were older (median age 54 years vs. 52 years in historical cohort, *p* < 0.001), more likely to receive allografts from younger donors (donor median age 32 years vs. 34 years in historical cohort, *p* = 0.005) and reduced‐intensity conditioning regimens (*p* = 0.001). The two cohorts also differed for type of employed donor and HSCs source, being unrelated donors (URD) and peripheral blood‐derived stem cells (PBSC) more used in the “cryo” cohort (*p* < 0.001), reporting a trend consistent with that observed in Europe [18]. Consequently, a greater use of in vivo T‐cell depletion in this cohort was registered (*p* < 0.001). Additionally, unfavorable donor/recipient CMV serostatus (patient negative with donor positive) was prominent in the “cryo” group (*p* < 0.001). As expected, taking into account different transplant eras, median follow‐up was 23 months (IQR: 18–28) for the “cryo” cohort and 41 months (IQR: 37–48) for the historical cohort. Separate clinical data for adults and pediatrics are provided in Tables S3 and S4.

**TABLE 1:** Patients' characteristics.

	Historical cohort *n* = 2516	Cryo cohort *n* = 976	*p*
Median age at allo‐HSCT, years (IQR)	52 (37–61)	54 (42–63)	**< 0.001**
Male/female, *n* (%)	1444 (57.4)/1072 (42.6)	579 (59.3)/397 (40.7)	0.303
Donor median age, years (IQR)	34 (25–45)	32 (25–42)	**0.005**
Diagnosis, *n* (%)			0.098
Acute leukemia	1472 (58.5)	585 (59.9)	
Lymphoma/multiple myeloma	419 (16.6)	166 (17.1)	
MDS/MPN and MPN	452 (18)	179 (18.3)	
Chronic leukemia	38 (1.5)	15 (1.5)	
Bone marrow failure	69 (2.7)	20 (2)	
Other^a^	66 (2.7)	11 (1.2)	
Disease status at allo‐HSCT			0.781
CR/not CR, *n* (%)	1528 (60.7)/835 (33.2)	612 (62.7)/327 (33.5)	
Missing, *n* (%)	153 (6.1)	37 (3.8)	
KPS, 90–100/ < 90, *n* (%)	1967 (78.7)/533 (21.3)	779 (80.4)/190 (19.6)	0.284
Myeloablative conditioning, *n* (%)	1807 (72.0)	647 (66.3)	**0.001**
Type of donor, *n* (%)			**< 0.001**
Haploidentical	754 (30.0)	219 (22.4)	
MRD	593 (23.6)	167 (17.1)	
URD	1168 (46.4)	590 (60.5)	
Source PBSCs, *n* (%)	1825 (72.6)	934 (95.7)	**< 0.001**
T‐cell depletion, *n* (%)	1696 (69.5)	760 (78.3)	**< 0.001**
Type of T‐cell depletion, *n* (%)			**0.003**
ATG	832 (49.1)	381 (50.1)	
PTCy	834 (49.2)	349 (45.9)	
ATG + PTCy	30 (1.8)	30 (4.0)	
At least 1 comorbidity, *n* (%)	1082 (43.3)	470 (48.4)	**0.008**
CMV donor/patient, *n* (%)			**< 0.001**
−/−	277 (11.2)	92 (9.5)	
−/+	659 (26.6)	332 (34.2)	
+/−	217 (8.8)	82 (8.4)	
+/+	1326 (53.5)	465 (47.9)	
Median follow‐up, months (IQR)	41 (37–48)	23 (18–28)	

*Note:* Bold *p* values are statistically significant (< 0.05).

Abbreviations: Allo‐HSCT, allogeneic hematopoietic stem cell transplantation; ATG, anti‐thymocyte globulin; CR, complete response; KPS, Karnofsky Performance Status; MDS/MPN, myelodysplastic/myeloproliferative neoplasms; MPN, myeloproliferative neoplasms; MRD, matched related donor; PBSC, peripheral blood‐derived stem cells; PTCy, post‐transplant cyclophosphamide; URD, unrelated donor.

^a^ Hemoglobinopathies (*n* = 38 in the historical and *n* = 3 in the cryo cohort, respectively), inherited disorders (*n* = 2 in the historical and *n* = 1 in the cryo cohort, respectively), primary immunodeficiencies (*n* = 20 in historical an *n* = 6 in cryo cohort, respectively), and familial erythrophagocytic and familial hemophagocytic lymphohistiocytosis (FELH/FHLH) (*n* = 6 in historical and *n* = 1 in cryo cohort, respectively).

### 3.2 Cryopreserved Allografts

Median transit time, as previously described, was 1 day (IQR 0–2). Graft manipulation was reported in 506 cases (51.8%) of the patients belonging to the cryo cohort, with deplasmation (*n* = 348) being the most frequent, followed by erithrodepletion (*n* = 40) and dimethyl sulfoxide (DMSO) removal (*n* = 35). Of note, only one case of non‐compliant product due to post‐thaw CD34+ viability < 30% was recorded. Data related to the cryopreserved allografts are described in Table S2. Forty‐seven patients (5%) experienced adverse events during infusion (Figure S1), with only 2 cases of grade 3 post‐infusion desaturation episodes, as defined by the Common Terminology Criteria for Adverse Events (CTCAE).

### 3.3 Hematopoietic Engraftment and Graft Failure

At univariable analysis (Table 2), cumulative incidence of neutrophil engraftment at day 30 (30‐day CI) was similar between the “cryo” cohort (90.9% [95% CI: 88.9%–92.6%]) and historical group (91.8% [95% CI: 90.7%–92.9%]) with median time to engraftment of 18 days in both groups (IQR: 16–22 and 15–21, respectively). Conversely, 30‐day cumulative incidence of platelet recovery appeared to be affected by cryopreservation (71.1% [95% CI 68.1–73.9] and 78.3% [95% CI 76.6–79.9], respectively), while median time to engraftment was not different (19 days (IQR: 14–27) in the “cryo” cohort and 18 days (IQR: 13–25) in the historical cohort).

**TABLE 2:** Cryopreservation effect on study endpoints: Summary of univariable and multivariable analyses.

Outcome^a^	Univariable analysis			Multivariable analysis		
Cumulative incidence	sHR	95% CI	*p*	sHR	95% CI	*p*
Neutrophil engraftment	0.9	0.8–1.0	0.068	0.8	0.7–1.0	0.033
Platelet engraftment	0.8	0.7–0.9	< 0.001	0.7	0.6–0.8	< 0.001
Grade II‐IV aGVHD	1.2	1.0–1.4	0.152	1.2	0.9–1.5	0.194
Grade III‐IV aGVHD	1.2	0.9–1.7	0.210	1.1	0.8–1.6	0.589
cGVHD						
Patients < 18 years	4.5	2.1–9.5	< 0.001	3.9	1.7–9.1	0.002
Patients 18–55 years	0.7	0.6–0.9	0.006	0.7	0.5–0.9	0.008
Patients > 55 years	0.9	0.7–1.1	0.176	0.8	0.6–1.1	0.144
Extensive cGVHD	0.8	0.6–1.1	0.185	0.8	0.6–1.1	0.165
Cumulative incidence of relapse	1.0	0.9–1.1	0.814	1.0	0.9–1.2	0.943
Non‐relapse mortality	1.2	1.0–1.4	0.050	1.1	1.0–1.3	0.197
Relapse‐free survival	1.1	1.0–1.3	0.178	1.1	1.0–1.2	0.174
Overall survival	1.2	1.0–1.4	0.052	1.2	1.0–1.3	0.038

Abbreviations: aGVHD, acute GVHD; cGVHD, chronic GVHD.

^a^ Reported models according to age class when interaction was significant.

At multivariable analysis (Table 2), graft cryopreservation did impact on day30‐incidence of neutrophil and platelet engraftment (adjusted sHR = 0.8 [95% CI: 0.7–1], *p* = 0.033 and adjusted sHR = 0.7 [95% CI: 0.6–0.8]; *p* < 0.001, respectively). The adjusted 30‐day CI of neutrophil engraftment was 90.1% (95% CI: 88.4%–91.8%) in the cryopreserved cohort and 93.4% (95% CI: 92.4%–94.4%) in the historical cohort (*p* = 0.03). Similarly, the adjusted 30‐day CI of platelet engraftment was 68.2% (95% CI: 79.5%–83.1%) in the cryopreserved cohort and 68.2% (95% CI: 65.4%–71.0%) in the non‐cryopreserved group.

Nevertheless, the rate of primary graft failure at day 30 after allo‐HSCT in patients who received cryopreserved grafts mirrored that of the historical cohort in the primary analysis (5%, 95% CI: 3.7%–6.6% vs. 4%, 95% CI: 3.3%–4.9%; *p* = 0.337) and in the sensitivity analysis (9.1% [95% CI 7.4–11.2] vs. 7.8% [95% CI 6.8–9], respectively; *p* = 0.321), also after adjustment for other variables (adjusted RR = 1.19, 95% CI: 0.70–2.03, *p* = 0.518 in the primary analysis and adjusted RR = 1.13, 95% CI: 0.82–1.54, *p* = 0.542 in the sensitivity analysis, respectively).

Separated analyses for adults and pediatrics are provided in Tables S5 and S6.

### 3.4 aGVHD and cGVHD Cumulative Incidence

**FIGURE 1:**
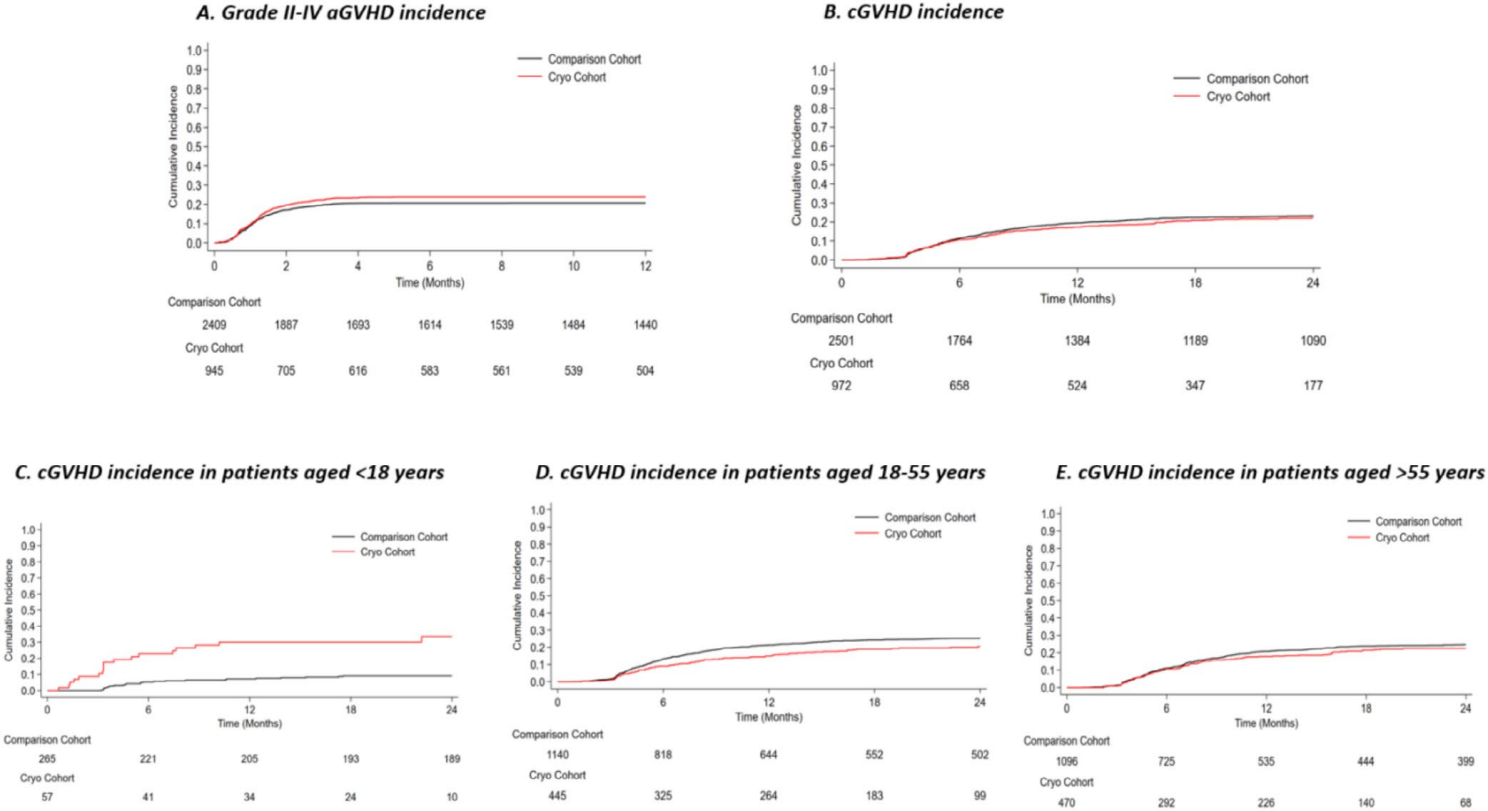
Acute and chronic GVHD incidence in the two cohorts. (A) Cumulative incidence of grade 2–4 aGVHD: 30‐day cumulative incidence (CI) 9.6% (95% CI: 7.4–11.1) in cryo cohort versus 9.2% (95% CI: 8.1–10.4) in historical cohort, 100‐day CI 23.1% (20.5%–25.9%) and 20.3% (95% CI: 18.7–21.9), respectively; (B) Cumulative incidence of cGVHD: 1‐year CI 17.3% (95% CI: 14.9–19.7) in cryo cohort versus 19.5% (95% CI: 18–21.1) in historical cohort, 2‐year CI 22.2% (95% CI: 19.5–25) and 23.3% (95% CI: 21.6–24.9), respectively; (C) Cumulative incidence of cGVHD in patients aged < 18 years: 1‐year CI 30% (95% CI: 18.7%–42.1%) in cryo cohort versus 7.2% (95% CI: 4.5%–10.7%) in historical cohort and 2‐year CI 33.6% (95% CI: 20.9%–46.8%) in cryo cohort versus 9.1% (95% CI: 6%–13%) in historical cohort; (D) Cumulative incidence of cGVHD in patients aged 18–55 years: 1‐year CI 15% (95% CI: 11.8%–18.5%) in cryo cohort versus 21.3% (95% CI: 19%–23.8%) in historical cohort and 2‐year CI 20.5% (95% CI: 16.6%–24.6%) in cryo cohort versus 25.3% (95% CI: 22.8%–27.8%) in historical cohort; (E) Cumulative incidence of cGVHD patients aged > 55 years: 1‐year CI: 17.9% (95% CI: 14.5%–21.5%) in cryo cohort versus 20.7% (95% CI: 18.4%–23.2%) in historical cohort and 2‐year CI 22.4% (95% CI: 18.6%–26.4%) in cryo cohort versus 24.7% (95% CI: 22.1%–27.3%) in historical cohort. [Color figure can be viewed at wileyonlinelibrary.com]

At univariable analysis, cumulative incidence of grade II‐IV aGVHD at day 100 after allo‐HSCT was superimposable between the two cohorts (Figure 1A, Table 2). Similarly, 100‐day cumulative incidence of grade III‐IV aGVHD was comparable in both groups (8.4% [95% CI: 6.7%–10.2%] in the cryo vs. 6.9% [95% CI: 5.9%–7.9%] in the historical cohort). At multivariable analysis, risk of grade II‐IV aGVHD and grade III‐IV aGVHD was not statistically different between the two groups (Table 2).

The crude 1‐year and 2‐year cumulative incidence of cGVHD were similar between the cryo group and the historical cohort (Figure 1B). At univariable analysis (Table 2), cryopreservation was associated with different cGVHD cumulative incidence according to age class (Figure 1C–E). Indeed, a higher cGVHD incidence was observed among patients aged < 18 years (*p* < 0.001) in the cryo cohort compared to the historical cohort, while a lower incidence was reported among “cryo” adult patients (aged 18–55 years) (*p* = 0.006). Patients aged > 55 years in the cryo cohort had the same risk of cGVHD as the historical cohort at univariable analysis. Multivariable analysis (Table 2) confirmed such differences (patients aged < 18 years: adjusted sHR = 3.9, 95% CI: 1.7%–9.1%, *p* = 0.002; patients aged 18–55 years: adjusted sHR = 0.7, 95% CI: 0.5–0.9, *p* = 0.008; patients aged > 55 years: adjusted sHR = 0.8, 95% CI: 0.6–1.1, *p* = 0.144). 1‐year cumulative incidence of extensive cGVHD was 7.1% (95% CI: 5.6%–8.9%) in the cryo group and 8.6% (95% CI: 7.5%–9.7%) in the historical cohort (*p* = 0.433) and did not differ also after adjustment at multivariable analysis (Table 2).

Separated analyses for adults and pediatrics are provided in Tables S5 and S6.

### 3.5 Cumulative Incidence of Relapse, Non‐Relapse Mortality, Relapse‐Free Survival, and Overall Survival

**FIGURE 2:**
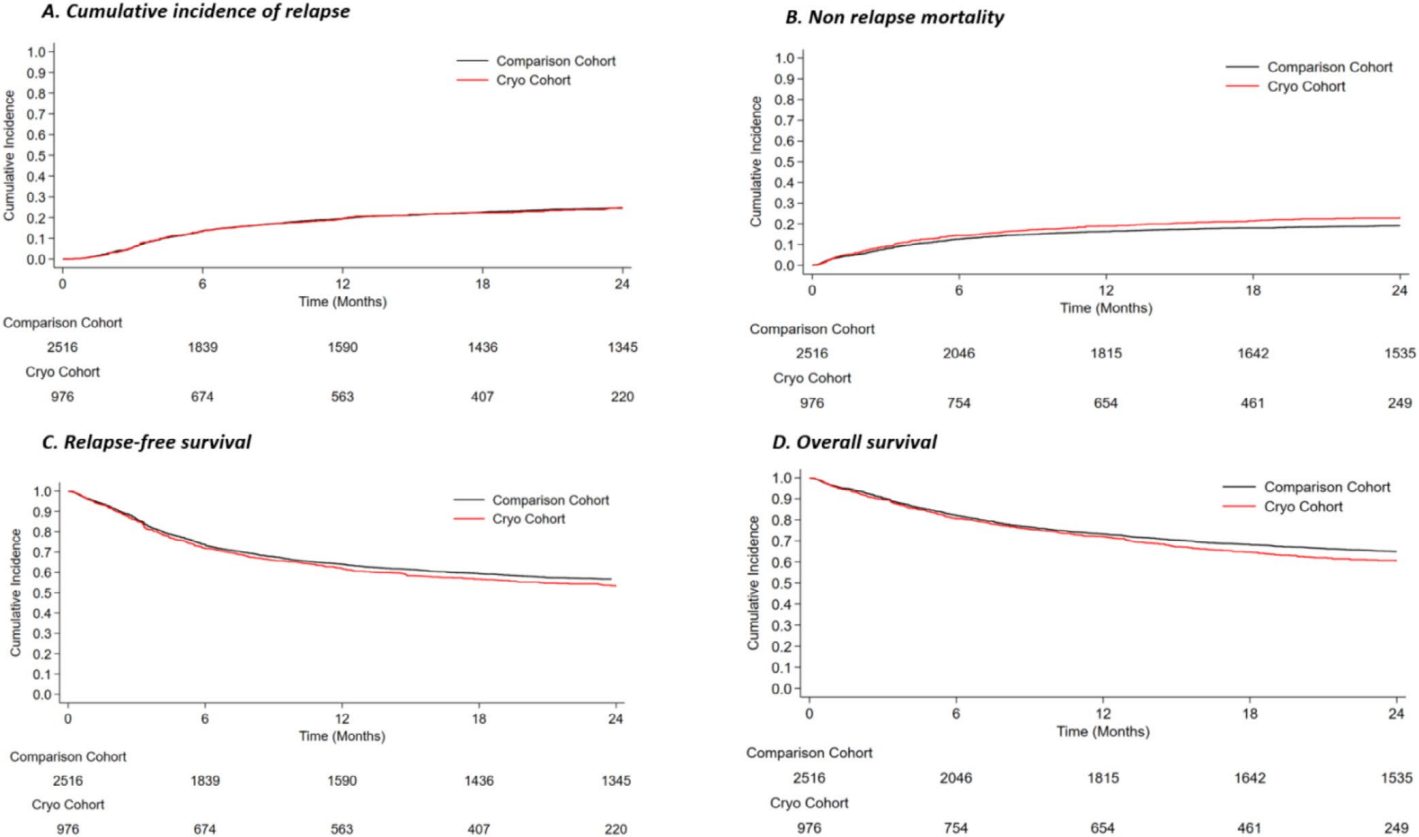
Post‐transplant outcomes incidence in the two cohorts. (A) Cumulative incidence of relapse: 1‐year CI 19.5% (95% CI: 17.0%–22.1%) in cryo cohort and 19.5% (17.9%–21.0%) in historical cohort; 2‐year CI 24.8% (95% CI: 21.9%–27.7%) in cryo cohort and 24.6% (95% CI: 22.9%–26.3%) in historical cohort; (B) Non‐relapse mortality: 1‐year CI 18.9% (95% CI: 16.5–21.5) in cryo cohort versus 16.3% (95% CI: 14.8–17.8) in historical cohort, 2‐year CI 22.9% (95% CI: 20.225.8) versus 19% (95% CI: 17.5–20‐6), respectively; (C) Relapse‐free survival: At 1 year 61.9% (95% CI: 58.7–64.9) in cryo cohort versus 64% (95% CI: 62.1 65.8) in historical cohort; at 2 years 53.2% (95% CI: 49.8–56.5) versus 56.7% (95% CI: 54.7–58.6), respectively; (D) Overall survival: At 1 year 72% (95% CI: 69–74.8) in the cryo versus 73.3% (71.5–74.9) in the historical cohort; at 2 years 60% (95% CI: 57–63.7) versus 65% (95% CI: 63–66.8), respectively. [Color figure can be viewed at wileyonlinelibrary.com]

There was no difference in cumulative incidence of relapse between the two cohorts (Figure 2A) at univariable analysis, and the risk of relapse remained similar also after adjustment at multivariable analysis (Table 2). NRM showed a trend toward a greater risk in the cryo cohort (sHR 1.2 [95% CI 1–1.4], *p* = 0.050, Figure 2B). However, after adjustment, cryopreservation did not impact NRM (Table 2). A significantly higher number of deaths due to infection was registered in the cryo cohort (*n* = 106 [29.4%] vs. *n* = 231 [23.7%] in the historical cohort; *p* = 0.040).

“Cryo” patients showed similar outcomes in terms of RFS compared to the historical cohort (Figure 2C) at univariable and multivariable analysis (Table 2). Patients who received cryopreserved allografts displayed a trend toward shorter OS at univariable (HR = 1.2, 95% CI: 1.0–1.4, *p* = 0.052), which was confirmed in multivariable analysis (adjusted HR = 1.2, 95% CI: 1.0–1.3, *p* = 0.038) (Figure 2D, Table 2).

Separated analyses for adults and pediatrics are provided in Tables S5 and S6.

### 3.6 Impact of Transit Time on Primary and Secondary Endpoints in the Study Cohort

Patients' characteristics according to transit time (< 2 days vs. ≥ 2 days) are described in Table S7. We found that donor age was significantly lower, and there was a higher prevalence of unrelated donors for products cryopreserved ≥ 2 days from collection. Additionally, this category showed a lower number of CD34+ cells after thawing compared to those cryopreserved earlier (transit time < 2 days) (median 5 × 10^6^/kg, IQR 3.7–7 vs. 6 × 10^6^/kg, IQR 4.3–7.6, respectively; *p* = 0.005).

Results of univariable and multivariable analyses are reported in Table 3. At multivariable analysis, there was no significant difference in terms of neutrophil and platelet engraftment between patients with graft transit time < 2 days and those with longer transit time. Cumulative incidence of grade II‐IV aGVHD, grade III‐IV aGVHD, cGVHD, and extensive cGVHD appeared to be similar in the two groups at uni‐ and multivariable analysis. Cumulative incidence of relapse was higher among patients with transit time ≥ 2 days at uni‐ and multivariable analysis (adjusted sHR = 1.61, 95% CI 1.08–2.40, *p* = 0.020). No difference was found in terms of NRM at uni‐ and multivariable analysis. Relapse‐free survival showed to be different according to type of donor (*p* = 0.007). Specifically, “cryo” patients who received allo‐HSCT from MRD or haploidentical donor with transit time ≥ 2 days had an inferior RFS at uni‐ and multivariable analysis (adjusted HR = 2.17, 95% CI 1.57–3.00, *p* < 0.001). Conversely, patients who received URD did not display different RFS according to transit time. Overall survival was also different according to type of donor. Indeed, “cryo” patients who underwent allo‐HSCT from MRD or haploidentical donor with transit time ≥ 2 days showed a shorter OS at uni‐ and multivariable analysis (adjusted HR = 1.79, 95% CI 1.08–2.96, *p* = 0.023). No difference was observed among patients who received cryopreserved allografts from URD.

**TABLE 3:** Impact of transit time (< 2 days and ≥ 2 days) in the “cryo” cohort at univariable and multivariable analysis.

			Univariable analysis			Multivariable analysis		
Outcome	Cumulative incidence % (95% CI)		sHR	95% CI	p	sHR	95% CI	*p*
Neutrophil engraftment (30‐day)	Transit time < 2 days	89.9% (87.3%–91.9%)	—	—	—	—	—	—
	Transit time ≥ 2 days	93.1% (89.5%–95.5%)	1.27	1.02–1.57	0.033	1.17	0.93–1.46	0.186
Platelet engraftment (30‐day)	Transit time < 2 days	70.5% (66.8%–73.9%)	—	—	—	—	—	—
	Transit time ≥ 2 days	72.1% (66.5%–76.9%)	1.06	0.89–1.26	0.503	0.87	0.72–1.06	0.158
Grade II–IV aGVHD (100‐day)	Transit time < 2 days	22.5% (19.4%–25.8%)	—	—	—	—	—	—
	Transit time ≥ 2 days	24.6% (19.8%–29.7%)	1.09	0.81–1.46	0.589	1.25	0.87–1.80	0.228
Grade III–IV aGVHD (100‐day)	Transit time < 2 days	8.1% (6.2%–10.4%)	—	—	—	—	—	—
	Transit time ≥ 2 days	9.0% (6.0%–12.6%)	1.08	0.69–1.69	0.737	1.39	0.77–2.52	0.278
cGVHD (2 years)	Transit time < 2 days	22.0% (18.8%–25.3%)	—	—	—	—	—	—
	Transit time ≥ 2 days	21.8% (16.9%–27.1%)	0.93	0.73–1.18	0.548	0.98	0.74–1.31	0.903
extensive cGVHD (2 years)	Transit time < 2 days	9.4% (7.3%–11.8%)	—	—	—	—	—	—
	Transit time ≥ 2 days	8.6% (5.5%–12.6%)	0.84	0.55–1.29	0.433	0.86	0.56–1.31	0.479
Cumulative incidence of relapse (2 years)	Transit time < 2 days	21.9% (18.6%–25.3%)	—	—	—	—	—	—
	Transit time ≥ 2 days	31.7% (26.0%–37.4%)	1.59	1.19–2.14	0.002	1.61	1.08–2.40	0.020
Non‐relapse mortality (2 years)	Transit time < 2 days	24.3% (21.0%–27.8%)	—	—	—	—	—	—
	Transit time ≥ 2 days	20.3% (15.7%–25.3%)	0.82	0.63–1.06	0.135	0.88	0.64–1.23	0.459

	Survival (95% CI)		HR	95% CI	*p*	HR	95% CI	*p*
Relapse‐free survival^a^ (2 years)								
MRD/Haplo	Transit time < 2 days	56.0% (50.6%–61.2%)	—	—	—	—	—	—
	Transit time ≥ 2 days	25.0% (6.0%–50.5%)	2.19	1.43–3.36	< 0.001	2.41	1.57–3.00	< 0.001
URD	Transit time < 2 days	53.9% (47.5%–59.8%)	—	—	—	—	—	—
	Transit time ≥ 2 days	49.5% (43.0%–55.6%)	1.20	0.93–1.57	0.166	1.21	0.90–1.63	0.196
Overall survival^a^ (2 years)								
MRD/Haplo	Transit time < 2 days	59.6% (54.0%–64.7%)	—	—	—	—	—	—
	Transit time ≥ 2 days	33.3% (10.3%–58.8%)	2.01	1.44–2.81	< 0.001	1.79	1.08–2.96	0.023
URD	Transit time < 2 days	62.9% (56.8%–68.4%)	—	—	—	—	—	—
	Transit time ≥ 2 days	59.8% (53.3%–65.8%)	1.08	0.85–1.37	0.529	1.14	0.88–1.49	0.319

Abbreviations: aGVHD, acute GVHD; cGVHD, chronic GVHD; Haplo, haploidentical donor; MRD, matched related donor; URD, unrelated donor.

^a^ Reported multivariate models according to donor type when tested for interaction with transit time were significant.

## 4 Discussion

Cryopreservation has become an attractive option in many transplant centers, providing greater flexibility in the timing and coordination of donor collection and allo‐HSCT. However, the safety and comparability of cryopreserved grafts to fresh products have yet to be fully clarified. Our study aimed to evaluate the characteristics and safety of cryopreserved transplants and to assess their implications for transplant outcomes.

The first important finding of this experience is that cryopreservation of allografts is feasible, with only one case of significant loss of viability after the procedure. Despite a relatively low rate of DMSO removal (reported in less than 10% of cases) and other manipulations, typically mild to moderate adverse events were reported during infusion, thus supporting the safety and quality of stem cell cryopreservation [19].

As expected, PBSC source was more common in the cryopreserved cohort leading to an increased use of T‐cell depletion. This trend may be attributed to the preference for PBSC since the onset of the COVID‐19 pandemic, as it allows for faster recovery and easier collection and helps prevent stem cell loss during cryopreservation [14].

However, despite the prevalent use of PBSCs, the cumulative incidence of engraftment at day 30 after allo‐HSCT was diminished by cryopreservation, as reported in most studies [4, 6, 9, 20].

The largest available study by the Center for International Blood and Marrow Transplant Research (CIBMTR) [5], including roughly 4000 subjects, showed a decreased 30‐day incidence of neutrophil and platelet recovery among the recipients of cryopreserved allografts compared to the fresh group (95.4% vs. 96.7%, respectively, *p* = 0.041; 71.8% vs. 78.1%, *p* < 0.001, respectively). While this study noted an increased risk of primary graft failure in the cryopreserved cohort, our findings showed that the rate of primary graft failure was comparable between the two groups. Such a finding could be related to the higher use of T‐cell depletion in the cryo cohort compared to the historical cohort, as shown by other studies [7, 11, 12, 21, 22, 23]. Additionally, concerns have been raised about the time from collection date to cryopreservation date (transit time). Historical data suggest that prolonged transit time before freezing could compromise CD34+ cell counts and viability, potentially leading to delayed engraftment and worse outcomes [2, 24]. However, in our experience, despite a decline in CD34+ cells number, transit time did not significantly affect hematological recovery or increase the risk of graft failure. This outcome emphasizes the efficiency of Italian stem cell laboratories in delivering high‐quality products, contributing to the reduction of risks associated with graft cryopreservation and manipulation [25].

Regarding the effect of cryopreservation on GVHD, we found no significant difference in the cumulative incidence of grade II‐IV and grade III‐IV aGVHD with the use of cryopreserved grafts, consistent with findings from several studies [3, 4, 5, 6, 11, 12, 20, 21]. In parallel, the incidence of cGVHD exhibited a bimodal pattern, being higher among younger “cryo” patients (aged < 18 years) and lower among adult “cryo” patients (aged 18–55 years), while no significant difference was observed in patients aged > 55 years between the two groups. A clear explanation for this bidirectional effect is difficult to determine. Some studies in the literature suggest that cryopreserved grafts may reduce the incidence of cGVHD by favoring the selection of the T‐regulatory (T‐reg) subset [3, 5, 10]. Additionally, a reduction in cGVHD has been documented in pediatric transplant populations using post‐transplant cyclophosphamide (PTCy) as GVHD prophylaxis [3]. In our context, the use of PTCy in pediatric patients of the cryo cohort was very limited compared to the historical cohort (5.4% vs. 28.9%, respectively), as Italian pediatric transplant physicians tended to reserve its use for selected cases due to concerns about potential late adverse effects. Instead, alpha‐beta ex vivo T‐cell depletion is widely applied, particularly in the setting of non‐malignant diseases [26, 27]. The shift in transplant paradigms during the pandemic may have further influenced these results.

The impact of graft cryopreservation on immune cells could also lead to an impaired graft‐versus‐leukemia effect, as reported by some authors [2, 10]. Clinically, this has been associated with an increased incidence of relapse and inferior relapse‐free survival in a few studies [3, 5, 9]. In our study, we did not observe any difference in the cumulative incidence of relapse or RFS between the cryopreserved cohort and the historical cohort.

Indeed, we observed a trend toward higher NRM among patients receiving cryopreserved grafts, though this was not confirmed in multivariable analysis. However, a shorter OS was noted in this group. These findings may be attributed to the reduced incidence and delayed hematopoietic recovery and a higher rate of infections, which were significantly more frequent causes of death among patients receiving cryopreserved transplants. Unfortunately, we lacked data on the precise causes of infection or on immune reconstitution in the two patient groups, making it impossible to definitively establish the actual effect of cryopreservation on immune recovery. However, several reports have documented the effects of cryopreservation on both the number and function of immune cells [28]. Additionally, the SARS‐CoV‐2 pandemic may have significantly contributed to a higher post‐transplant infectious risk in the more recent cryo cohort, given the vulnerability of these patients and the constraints faced by the healthcare system during this period [29].

Notably, a significant increase in relapse risk was observed when the time between HSCs collection and cryopreservation exceeded 1 day. Additionally, shorter OS and RFS were noted in MRD and haploidentical transplants, though not in URD transplants, depending on transit time.

This suggests that graft age at cryopreservation may affect lymphocyte function and the likelihood of achieving full donor chimerism, emphasizing the importance of minimizing transit time to maintain optimal graft quality, particularly in high‐risk transplants such as haploidentical settings. These differences might be partially attributed to variations in graft quality and donor age, which is typically younger in URD donors compared to MRD and haploidentical transplants, as observed in our study and others [30].

These findings could inform clinical decision‐making regarding the use of cryopreserved grafts based on patient‐, donor‐, and disease‐specific risk factors. For instance, a fresh graft might be preferable in cases of active neoplastic disease, especially when the donor is overseas, while cryopreserved grafts could be considered for patients in first remission, balancing the procedure's risks and benefits.

We acknowledge several limitations of our study, including its retrospective nature and the potential for some patients in the historical cohort to have received cryopreserved grafts. However, as reported in a recent EBMT survey, only 7.4% of transplant centers used cryopreserved HPC products before the pandemic [13]. Despite these limitations, the inclusion of a large cohort of transplanted patients within the robust Italian GITMO network over a recent and relatively short timeframe allows for meaningful observations.

Our findings support the general safety of cryopreservation, which remains a viable option when safety or logistical constraints could otherwise hinder the transplant process. However, caution is warranted with the use of cryopreserved products, especially when the interval between collection and cryopreservation is prolonged, given their potential impact on relapse risk and OS. Transplant centers should carefully balance the benefits and drawbacks of cryopreservation, considering its clinical implications and the potential for immunological dysfunction.

## Ethics Statement

Each patient provided consent for the collection of data within the GITMO/EBMT registry.

## Conflicts of Interest

The authors declare no conflicts of interest.

## Supporting information


**Data S1.** Supporting Information.

## Data Availability

The data that support the findings of this study are available from the corresponding author upon reasonable request.
