# Supplementary material for: Clinical Impact of Graft Cryopreservation on Allogeneic Stem Cell Transplantation: An Italian, Registry‐Based Study on Behalf of the “Gruppo Italiano Per Il Trapianto di Midollo Osseo, Cellule Staminali Emopoietiche e Terapia Cellulare” (GITMO)
Source: Am J Hematol. 2025 Jun 2;100(8):1354–64. doi: 10.1002/ajh.27731 (PMC12232522; doi:10.1002/ajh.27731)
Supplement: Supplementary file 1 — Data S1. Supporting Information. [file AJH-100-1354-s001.docx]

**Supplementary Figures**

**Supplementary Figure 1. Adverse events during infusion reported in 47 (5%) patients of the cryo cohort**

**Supplementary Figure 2. Causes of death in the two cohorts.**

**
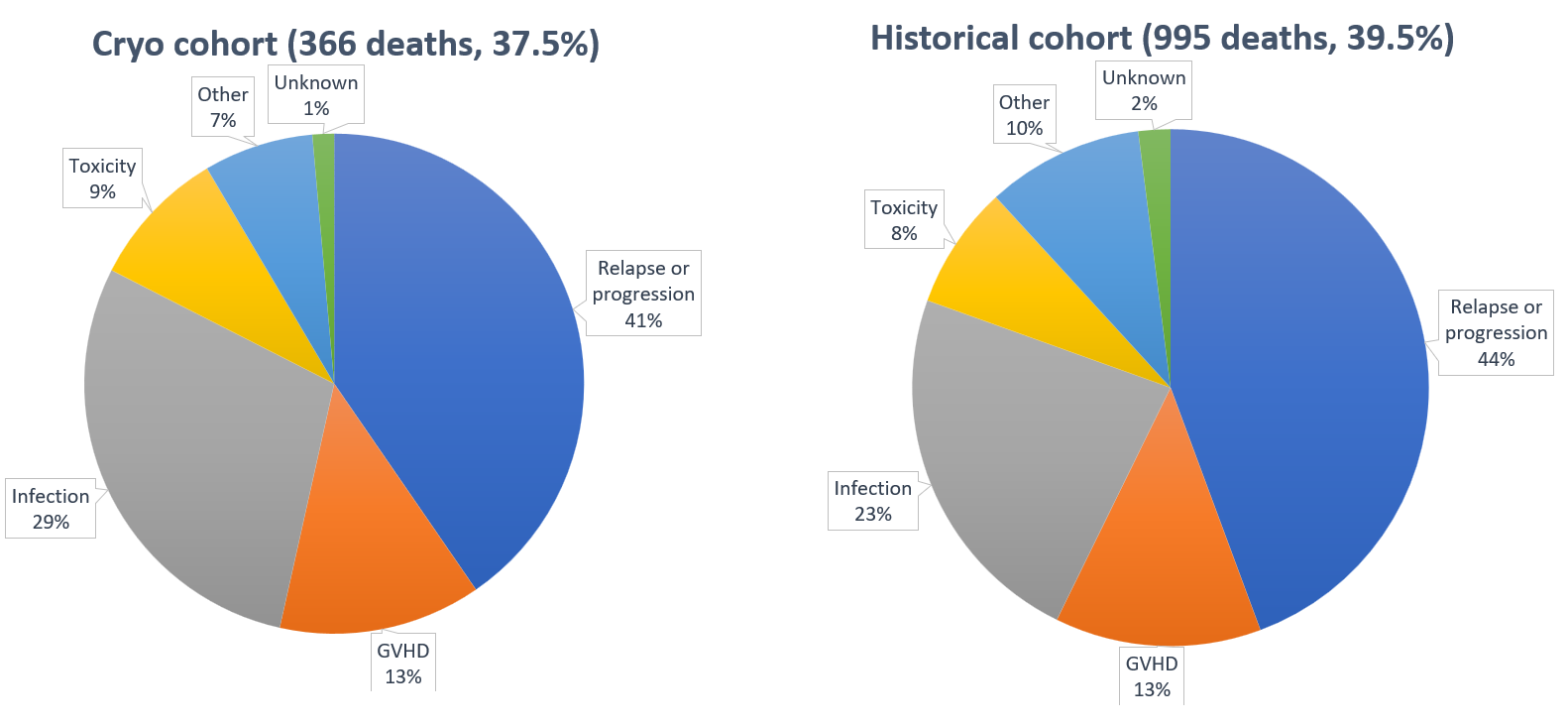
**

**Supplementary Tables**

**Supplementary Table 1. Centers participating in the study**

| **CIC** | **Centre** | **BMT Program Director** |
| --- | --- | --- |
| 825 | Alessandria | Marco Ladetto |
| 788 | Ancona | Attilio Olivieri |
| 119 | Ascoli Piceno | Pietro Galieni |
| 789 | Avellino | Antonio Maria Risitano |
| 649 | Bari | Paola Carluccio |
| 790 | Bologna | Arcangelo Prete |
| 299 | Bolzano-Trento | Irene Cavattoni |
| 141 | Brescia | Domenico Russo |
| 920 | Brindisi | Domenico Pastore |
| 792 | Catania | Giuseppe Milone |
| 606 | Cuneo | Nicola Mordini |
| 304 | Firenze | Chiara Nozzoli |
| 1003 | Firenze | Veronica Tintori |
| 217 | Genova | Emanuele Angelucci |
| 274 | Genova | Maura Faraci |
| 354 | Milano | Stefania Bramanti |
| 294 | Milano | Roberto Cairoli |
| 813 | Milano | Fabio Ciceri |
| 265 | Milano | Francesco Onida |
| 543 | Modena | Angela Cuoghi |
| 544 | Monza | Matteo Parma |
| 607 | Napoli | Alessandra Picardi |
| 341 | Napoli | Francesco Paolo Tambaro |
| 766 | Napoli | Fabrizio Pane |
| 285 | Padova | Alessandra Biffi |
| 392 | Palermo | Luca Castagna |
| 692 | Palermo | Maurizio Musso |
| 245 | Parma | Lucia Prezioso |
| 1006.1 | Pavia | Marco Zecca/Nicola Polverelli |
| 248 | Pescara | Stella Santarone |
| 163 | Piacenza | Daniele Vallisa |
| 795 | Pisa | Fabio Benedetti |
| 587 | Reggio Calabria | Massimo Martino |
| 307 | Roma | Simona Sica |
| 232 | Roma | Anna Paola Iori |
| 928 | Salerno | Carmine Selleri |
| 321 | Siena | Monica Tozzi |
| 231 | Torino | Alessandro Busca |
| 305 | Torino | Franca Fagioli |
| 652 | Tricase | Vincenzo Pavone |
| 705 | Udine | Renato Fanin |
| 502 | Venezia | Cristina Skert |
| 623.2 | Verona | Simone Cesaro |
| 797 | Vicenza | Carlo Borghero |

**Supplementary Table 2. Characteristics of cryopreserved allografts**

| **Parameter (pre and post cryopreservation)** | **Before cryopreservation** | **Post-thaw** |
| --- | --- | --- |
| TNC x10^8^/kg, median (IQR) [missing] | 8 (6-11) [77] | 7 (5-10) [425] |
| CD34+ x10^6^/kg, median (IQR) [missing] | 7 (5-9) [26] | 6 (4-8) [381] |
| CD3+ x10^6^/kg, median (IQR) [missing] | 208 (144-292) [133] | 185 (121-266) [695] |
| CD34+ viability, median (IQR) [missing] | 99 (97-100) [217] | 91 (79-96) [163] |

**Supplementary Table 3. Adults’ characteristics in the two cohorts**

|  | **Historical cohort n=2250** | **Cryo cohort n=919** | **p-value** |
| --- | --- | --- | --- |
| **Median age at allo-HSCT, years (IQR)** | 55 (44-62) | 55 (45-63) | 0.096 |
| **Male/Female, n (%)** | 1,300 (57.8)/950 (42.2) | 540 (58.8)/379 (41.2) | 0.630 |
| **Donor median age, years (IQR)** | 35 (26-46) | 33 (25-43) | **<0.001** |
| **Diagnosis, n (%)** |  |  | 0.880 |
| Acute Leukemia | 1,341 (59.6) | 552 (60.1) |  |
| Lymphoma/Multiple Myeloma | 412 (18.3) | 164 (17.8) |  |
| MDS/MPN & MPN | 418 (18.6) | 176 (19.2) |  |
| Chronic leukemia | 38 (1.7) | 15 ( 1.6) |  |
| Bone Marrow Failure | 34 (1.5) | 11 (1.2) |  |
| Other* | 7 (0.3) | 1 (0.1) |  |
| **Disease status at allo-HSCT** |  |  | 0.84 |
| **CR/not CR, n (%)** | 1,398 (63.7)/795 (36.3) | 579 (64.2)/323 (35.8) |  |
| **KPS, 90-100/<90, n (%)** | 1,740 (77.8)/497 (22.2) | 730 (79.5)/188 (20.5) | 0.30 |
| **Myeloablative conditioning, n (%)** | 1,565 (69.7) | 594 (64.6) | **0.006** |
| **Type of donor, n (%)** |  |  | **<0.001** |
| Haploidentical | 684 (30.4) | 214 (23.3) |  |
| MRD | 551 (24.5) | 165 (18.0) |  |
| URD | 1,015 (45.1) | 540 (58.8) |  |
| **Source PBSCs, n (%)** | 1,748 (77.7) | 897 (97.6) | **<0.001** |
| **T-cell depletion, n (%)** | 1,568 (71.1) | 723 (79.0) | **<0.001** |
| **Type of T-cell depletion, n (%)** |  |  | **0.003** |
| ATG | 745 (33.8) | 347 (37.9) |  |
| PTCy | 797 (36.1) | 347 (37.9) |  |
| ATG+PTCy | 26 (1.2) | 29 (3.2) |  |
| **At least 1 comorbidity, n (%)** | 1,057 (47.3) | 466 (50.9) | 0.071 |
| **CMV donor/patient, n (%)** |  |  | **0.002** |
| -/- | 245 (11.0) | 86 (9.4) |  |
| -/+ | 604 (27.2) | 311 (34.0) |  |
| +/- | 180 (8.1) | 73 (8.0) |  |
| +/+ | 1,194 (53.7) | 445 (48.6) |  |
| **Median follow-up, months (IQR)** | 41 (35-48) | 23 (18-28) |  |

**Hemoglobinopathies (n=2 in historical and n=1 in the cryo cohort, respectively), primary immunodeficiencies (n=5 in historical and n=0 in the cryo cohort, respectively) were included.*

*Abbreviations.* Allo-HSCT: allogeneic hematopoietic stem cell transplantation; MDS/MPN: myelodysplastic/myeloproliferative neoplasms; MPN: myeloproliferative neoplasms; CR: complete response; KPS: Karnofsky Performance Status; MRD: matched related donor; URD: unrelated donor; PBSC: peripheral blood-derived stem cells; ATG: anti-thymocyte globulin; PTCy: post-transplant cyclophosphamide.

**Supplementary Table 4. Pediatrics’ characteristics in the two cohorts**

|  | **Historical cohort n=266** | **Cryo cohort n=57** | **p-value** |
| --- | --- | --- | --- |
| **Median age at allo-HSCT, years (IQR)** | 8 (3-13) | 7 (5-11) | 0.89 |
| **Male/Female, n (%)** | 144 (54.1)/122 (45.9) | 39 (68.4)/18 (31.6) | 0.056 |
| **Donor median age, years (IQR)** | 28 (21-37) | 27 (23-36) | 0.79 |
| **Diagnosis, n (%)** |  |  | 0.43 |
| Acute Leukemia | 131 (49.2) | 33 (57.9) |  |
| Lymphoma/Multiple Myeloma | 7 (2.6) | 2 (3.5) |  |
| MDS/MPN & MPN | 34 (12.8) | 3 (5.3) |  |
| Bone Marrow Failure | 35 (13.2) | 9 (15.8) |  |
| Other* | 59 (22.2) | 10 (17.5) |  |
| **Disease status at allo-HSCT** |  |  | 0.12 |
| **CR/not CR, n (%)** | 130 (76.5)/40 (23.5) | 33 (89.2)/4 (10.8) |  |
| **KPS, 90-100/<90, n (%)** | 227 (86.3)/36 (13.7) | 49 (96.1)/2 (3.9) | 0.059 |
| **Myeloablative conditioning, n (%)** | 242 (91.7) | 53 (93.0) | 1.000 |
| **Type of donor, n (%)** |  |  | **<0.001** |
| Haploidentical | 70 (26.4) | 5 (8.8) |  |
| MRD | 42 (15.8) | 2 (3.5) |  |
| URD | 153 (57.7) | 50 (87.7) |  |
| **Source PBSCs, n (%)** | 77 (29.1) | 37 (64.9) | **<0.001** |
| **T-cell depletion, n (%)** | 128 (54.2) | 37 (66.1) | 0.13 |
| **Type of T-cell depletion, n (%)** |  |  | **0.003** |
| ATG | 87 (36.9) | 34 (60.7) |  |
| PTCy | 37 (15.7) | 2 (3.6) |  |
| ATG+PTCy | 4 (1.7) | 1 (1.8) |  |
| **At least 1 comorbidity, n (%)** | 25 (9.5) | 4 (7.1) | 0.80 |
| **CMV donor/patient, n (%)** |  |  | 0.065 |
| -/- | 32 (12.5) | 6 (10.7) |  |
| -/+ | 55 (21.5) | 21 (37.5) |  |
| +/- | 37 (14.5) | 9 (16.1) |  |
| +/+ | 132 (51.6) | 20 (35.7) |  |
| **Median follow-up, months (IQR)** | 44 (37-49) | 23 (18-26) |  |

**Hemoglobinopathies (n=36 in historical and n=2 in the cryo cohort, respectively), inherited disorders (n=2 in historical and n=1 in the cryo cohort, respectively), primary immunodeficiencies (n=15 in historical and n=6 in the cryo cohort, respectively), and familial erytrhro-phagocytic and familial hemophagocytic lymphohistiocytosis (FELH/FHLH) (n=6 in historical and n=1 in the cryo cohort, respectively)*

*Abbreviations.* Allo-HSCT: allogeneic hematopoietic stem cell transplantation; MDS/MPN: myelodysplastic/myeloproliferative neoplasms; MPN: myeloproliferative neoplasms; CR: complete response; KPS: Karnofsky Performance Status; CR: complete response; MRD: matched related donor; URD: unrelated donor; PBSC: peripheral blood-derived stem cells; ATG: anti-thymocyte globulin; PTCy: post-transplant cyclophosphamide

**Supplementary Table 5: Cryopreservation effect on study endpoints: summary of univariable and multivariable analyses in adult patients**

| ***Outcome*** | ***Univariable analysis*** | | | ***Multivariable analysis*** | | |
| --- | --- | --- | --- | --- | --- | --- |
| ***Cumulative incidence*** | **sHR** | **95% CI** | **P value** | **sHR** | **95%CI** | **P value** |
| **Neutrophil Engraftment** | 0.9 | 0.8-1.0 | 0.047 | 0.8 | 0.7-1.0 | 0.042 |
| **Platelet Engraftment** | 0.8 | 0.7-0.9 | <0.001 | 0.7 | 0.6-0.8 | <0.001 |
| **Grade II-IV aGVHD** | 1.2 | 1.0-1.4 | 0.124 | 1.2 | 0.9-1.5 | 0.218 |
| **Grade III-IV aGVHD** | 1.3 | 0.9-1.8 | 0.126 | 1.2 | 0.8-1.7 | 0.393 |
| **cGVHD** | 0.8 | 0.7-0.9 | 0.010 | 0.8 | 0.6-0.9 | 0.011 |
| **Extensive cGVHD** | 0.8 | 0.6-1.1 | 0.177 | 0.8 | 0.6-1.1 | 0.147 |
| **Cumulative incidence of relapse** | 1.0 | 0.8-1.1 | 0.766 | 1.0 | 0.9-1.2 | 0.795 |
| **Non-relapse mortality** | 1.2 | 1.0-1.4 | 0.047 | 1.1 | 1.0-1.3 | 0.157 |
| **Relapse-free survival** | 1.1 | 0.9-1.3 | 0.203 | 1.1 | 1.0-1.3 | 0.134 |
| **Overall survival** | 1.2 | 1.0-1.3 | 0.052 | 1.2 | 1.0-1.3 | 0.025 |

**Supplementary Table 6: Cryopreservation effect on study endpoints: summary of univariable and multivariable analyses in pediatric patients**

| ***Outcome*** | ***Univariable analysis*** | | | ***Multivariable analysis*** | | |
| --- | --- | --- | --- | --- | --- | --- |
| ***Cumulative incidence*** | **sHR** | **95% CI** | **P value** | **sHR** | **95%CI** | **P value** |
| **Neutrophil Engraftment** | 0.9 | 0.6-1.3 | 0.552 | 0.9 | 0.6-1.3 | 0.469 |
| **Platelet Engraftment** | 0.8 | 0.4-1.4 | 0.393 | 0.8 | 0.5-1.3 | 0.317 |
| **Grade II-IV aGVHD** | 1.5 | 0.7-3.3 | 0.273 | 1.3 | 0.6-2.9 | 0.550 |
| **Grade III-IV aGVHD** | 0.9 | 0.4-2.1 | 0.748 | 0.3 | 0.1-0.8 | 0.024 |
| **cGVHD** | 4.5 | 2.1-9.5 | <0.001 | 3.9 | 1.7-9.1 | 0.002 |
| **Extensive cGVHD** | 1.8 | 0.7-4.7 | 0.238 | 2.0 | 0.3-12.9 | 0.481 |
| **Cumulative incidence of relapse** | 0.9 | 0.6-1.4 | 0.572 | 0.9 | 0.4-1.9 | 0.695 |
| **Non-relapse mortality** | 0.7 | 0.2-2.7 | 0.561 | 0.5 | 0.1-2.8 | 0.431 |
| **Relapse-free survival** | 0.8 | 0.4-1.7 | 0.578 | 1.4 | 0.6-3.6 | 0.462 |
| **Overall survival** | 0.7 | 0.3-2.1 | 0.556 | 0.9 | 0.3-2.8 | 0.840 |

**Supplementary Table 7: Patients’ characteristics of the “cryo” cohort according to transit time**

|  | **Transit time <2 days**  **(n=672)** | **Transit time ≥2 days**  **(n=298)** | **p-value** |
| --- | --- | --- | --- |
| **Median age at allo-HSCT, years (IQR)** | 54 (41-63) | 55 (43-63) | 0.48 |
| **Male/Female, n (%)** | 392 (58.3)/280 (41.7) | 181 (60.7)/117 (39.3) | 0.524 |
| **Donor median age, years (IQR)** | 35 (26-46) | 28 (23-35) | **<0.001** |
| **Diagnosis, n (%)** |  |  | 0.840 |
| Acute leukemia | 398 (59.2) | 182 (61.1) |  |
| Lymphoma/Multiple myeloma | 115 (17.1) | 51 (17.1) |  |
| MDS/MPN & MPN | 128 (19.1) | 50 (16.7) |  |
| Chronic leukemia | 9 (1.3) | 6 (2) |  |
| Bone marrow failure | 13 (2) | 7 (2.4) |  |
| Other* | 9 (1.3) | 2 (0.7) |  |
| **KPS, 90-100/<90, n (%)** | 532 (79.9)/134 (20.1) | 243 (81.8)/54 (18.2) | 0.538 |
| **Myeloablative conditioning, n (%)** | 420 (62.5) | 223 (74.8) | **<0.001** |
| **Type of donor, n (%)** |  |  | **<0.001** |
| Haploidentical | 208 (31) | 9 (3) |  |
| MRD | 161 (24) | 3 (1) |  |
| URD | 303 (45.1) | 286 (96) |  |
| **Source PBSCs, n (%)** | 634 (94.3) | 295 (99) | **<0.001** |
| **T-cell depletion, n (%)** | 528 (79) | 227 (76.4) | 0.398 |
| **At least 1 comorbidity, n (%)** | 328 (49) | 138 (46.6) | 0.530 |
| **CMV donor/patient (%)** |  |  | **<0.001** |
| -/- | 57 (8.5) | 34 (11.4) |  |
| -/+ | 194 (29) | 138 (46.5) |  |
| +/- | 52 (7.8) | 30 (10.1) |  |
| +/+ | 365 (54.6) | 95 (32) |  |

**Hemoglobinopathies (n=3 in the group with transit time <2 days and n=0 in the group with longer transit time, respectively), inherited disorders (n=1 in the group with transit time <2 days and n=0 in the group with longer transit time, respectively), primary immunodeficiencies (n=15 in historical and n=6 in the cryo cohort, respectively), and familial erytrhro-phagocytic and familial hemophagocytic lymphohistiocytosis (FELH/FHLH) (n=1 in the group with transit time <2 n=0 in the group with longer transit time, respectively) were included.*

*Abbreviations.* Allo-HSCT: allogeneic hematopoietic stem cell transplantation; MDS/MPN: myelodysplastic/myeloproliferative neoplasms; MPN: myeloproliferative neoplasms; KPS: Karnofsky Performance Status; MRD: matched related donor; URD: unrelated donor; PBSC: peripheral blood-derived stem cells.
